# Determination of menstrual hygiene management and genital hygiene behaviors of students: A university example from Turkey

**DOI:** 10.1590/1980-220X-REEUSP-2024-0113en

**Published:** 2024-11-22

**Authors:** Aytül Hadımlı, Ayşenur Akan, Güzin Kardeş, Buket Akkurt, Birsen Karaca Saydam

**Affiliations:** 1 Ege University, Faculty of Health Science, Department of Midwifery, Izmir, Turkey.; 2 Republic of Turkey Ministry of Health, Altindag Family Health Center No 17, Izmir, Turkey.

**Keywords:** Menstruation, Students, Hygiene, Menstrual Hygiene Products, Menstruação, Estudantes, Higiene, Produtos de Higiene Menstrual, Menstruación, Estudiantes, Higiene, Productos para la Higiene Menstrual

## Abstract

**Objective:**

This study aimed to evaluate the use of menstrual hygiene products and genital hygiene practices among female university students.

**Method:**

A cross-sectional study design was employed. Data were collected using the Descriptive Information Form and the Genital Hygiene Behaviors Scale (GHBS). The Independent Samples t-test, ANOVA, and Post-hoc Tukey tests were used for data analysis.

**Results:**

Significant associations were found between GHBS scores and variables such as year in school, marital status, perceived income status, attending a health-related department, willingness to receive training in menstruation and genital hygiene (p < 0.05).

**Conclusion:**

It is recommended that regular educational programs, inter-institutional collaborations, and awareness-raising activities be implemented to promote students’ reproductive health behaviors. Health policies should also aim to improve young people’s access to menstrual products.

## RESUMO

**Objetivo:**

Este estudo teve como objetivo determinar como as meninas que estudavam em uma universidade usavam produtos de higiene menstrual e como eram suas comportamentos de higiene genital.

**Método:**

Trata-se de um estudo transversal. O Formulário de Informações Descritivas e a Escala de Comportamentos de Higiene Genital (ECHG) foram utilizados para a coleta de dados. Os dados foram analisados usando a amostra independente t-test, ANOVA, e o teste Tukey post-hoc, um teste de múltiplas comparações.

**Resultados:**

A relação entre as pontuações médias obtidas pelas estudantes a partir do ECHG e as variáveis como ano na escola, estado civil, situação de renda percebida, receber educação em um departamento relacionado à saúde, estar disposto a receber treinamento em menstruação e higiene genital foi estatisticamente significativa (p < 0.05).

**Conclusões:**

É recomendado que programas frequentes de formação, colaborações interinstitucionais e atividades que promovam a consciência, sejam aplicadas com o objetivo de munir os estudantes de comportamentos que melhorem a saúde reprodutiva e que se desenvolvam políticas que facilitem o acesso das jovens aos produtos menstruais.

## RESUMEN

**Objetivo:**

Este estudio tenía la finalidad de determinar cómo utilizaban los productos de higiene menstrual las adolescentes que estudiaban en una universidad y cómo eran sus comportamientos de higiene genital.

**Método:**

Este es un estudio transversal. Para la recolección de datos se utilizó Formulario de Información Descriptiva y la Escala de Comportamientos de Higiene Genital (ECHG).

**Resultados:**

La relación entre las puntuaciones medias que los alumnos obtuvieron en la ECHG y las variables año de escolarización, estado civil, nivel de ingresos percibidos, RECIBIR educación en un departamento relacionado con la salud, estar dispuestos a recibir formación sobre menstruación e higiene genital fue muy significativa desde los indicadores estadísticos arrojados (p < 0.05).

**Conclusión:**

Se recomienda realizar programas regulares de entrenamiento, colaboraciones interinstitucionales y actividades que aumenten la concientización para dotar a los estudiantes de comportamientos que mejoren la salud reproductiva y desarrollar políticas de salud que faciliten el desarrollo de los jóvenes acceso a productos menstruales.

## INTRODUCTION

Reproductive health was first broadly discussed at the International Conference on Population and Development (ICPD) in Cairo in 1994. It was defined not only as the absence of disease or infirmity relating to the reproductive system but also as the complete physical, mental, and social well-being in all functions of the reproductive system^(1)^. Puberty, the transition from childhood to adulthood, is a critical phase during which the foundation for female reproductive health is established^(2)^. During this time, healthy women experience regular changes known as the menstrual or reproductive cycle. Menstruation refers to the cyclical bleeding that occurs in women of reproductive age^(3)^. Menstrual hygiene encompasses the proper use of menstrual products, cleaning of the genital area, disposal of used menstrual items, and addressing other health-related needs during the menstrual cycle^(4)^. Genital hygiene, on the other hand, refers to maintaining, protecting, and promoting the health of the female urogenital system through hygienic practices^(5)^. Adequate menstrual and genital hygiene practices are crucial for women’s health, as improper practices can disrupt the vaginal flora and lead to various health issues^(6)^. Research shows that in many parts of the world, girls entering puberty often lack accurate knowledge about menstruation and hold misconceptions, as the adults surrounding them also lack knowledge about sexuality, reproduction, and menstruation and feel discomfort in discussing issues related to these topics^(7,8)^. Moreover, the lack of access to safe menstrual products or their incorrect use can lead to concerns about leaks and unpleasant odors. This, in turn, may cause genital and urinary tract infections, impacting students’ health, education, and even leading to school absenteeism and decreased academic performance^(9)^.

Despite Turkey’s status as a developing country, access to clean toilets and potable water is generally available, even in rural and suburban areas. Educational institutions across the country, from kindergartens to universities, provide separate restroom facilities for boys and girls. However, as in many developing countries, menstrual hygiene practices may still be inadequate, often due to a lack of essential supplies like toilet paper and soap. Furthermore, social stigma surrounding menstruation, rooted in privacy concerns and cultural taboos, can drive many girls to adopt unsafe hygiene practices^(2,3)^. In order to safeguard and improve women’s health, it is essential to provide education, counseling, and care on both menstrual and genital hygiene. With advances in technology, new menstrual hygiene products are continually introduced to the market. While international studies have investigated women’s use of these products, there are relatively few studies in Turkey that explore the relationship between menstrual product use and genital hygiene behaviors^(10,11,12)^. This study aims to assess the menstrual hygiene practices and genital hygiene behaviors of female university students in Turkey.

## METHODS

### Study Design

This study employed a quantitative, descriptive, cross-sectional design.

### Sample

The study population consisted of female students attending a state university. A convenience sampling method was used, and the sample size was calculated using a sampling method for a known population. The university has 22 faculties and vocational schools, six of which (Faculty of Medicine, Faculty of Dentistry, Faculty of Pharmacy, Faculty of Nursing, Faculty of Health Sciences and Vocational School of Health Services) offer health-related education. Other faculties cover fields such as engineering, education, theology, tourism, and economics. In 2022, there were 26,653 female students enrolled at the university. The sample size was determined as 379 using the Epi Info 6 StatCalc program, with a 95% confidence interval and a 50% incidence rate. A total of 1,172 students, who voluntarily participated by completing an online questionnaire via Google Forms, comprised the final study sample. The questionnaire included the Descriptive Information Form and the Genital Hygiene Behaviors Scale.

### Data Collection

*Descriptive Information Form:* This form, developed based on the literature, included 25 questions. Eight questions addressed the socio-demographic characteristics of the students, while 17 questions explored their menstrual and genital hygiene practices.

*Genital Hygiene Behaviors Scale (GHBS):* The GHBS, developed by Karahan in 2017^(13)^ assesses genital hygiene behaviors in women. It consists of 23 items divided into three subscales: “General Hygiene Habits” (items 1–12), “Menstrual Hygiene” (Items 13–20), and “Awareness of Abnormal Findings” (Items 21–23). The items in the GHBS are rated on 5-point Likert type scale ranging from 5 (strongly agree) to 1 (strongly disagree). Responses are rated on a 5-point Likert scale, ranging from 5 (strongly agree) to 1 (strongly disagree). The possible total GHBS score ranges from 23 to 115, with higher scores indicating more positive genital hygiene behaviors. The Cronbach’s alpha value was 0.80 for the overall GHBS, 0.70 for the General Hygiene subscale, 0.74 for the Menstrual Hygiene subscale, and 0.81 for the Awareness of Abnormal Findings subscale^(13)^.

Before initiating the study, permission was obtained from the deans of all faculties at the university. Data were collected between June 2022 and December 2022. The link to the data collection forms (Google Forms) was sent to the students’ university email addresses. Completing the survey took approximately 5-10 minutes.

### Data Analysis

The data were analyzed using SPSS (Statistical Package for Social Sciences) version 26.0. Descriptive statistics (frequency, percentage, arithmetic mean, and standard deviation) were used to analyze the data. The normality of the GHBS data distribution was checked using a Q-Q plot, confirming normal distribution. For data analysis, the Independent Samples t-test, ANOVA, and Post-hoc Tukey tests (a multiple comparison method) were applied. A p-value of less than 0.05 was considered statistically significant.

### Ethical Aspects

This study was approved by Ege University Scientific Research and Publication Ethics Committee (Approval date: May 31, 2022, Protocol No: 1509). Students were informed about the study’s objectives on the first page of the Google Form, and those who consented to participate proceeded to complete the questionnaire.

## RESULTS

The mean age of the students participating in the study was 21.01 ± 3.03 years (min: 17, max: 50). Among the participants, 98.0% were single, 48.0% perceived their financial situation as “income equal to expenses,” and 43.8% received a scholarship. Additionally, 35.3% were first-year students, and 61.5% were enrolled in health-related departments.

Regarding menstruation, the mean age of menarche was 12.90 ± 1.31 years (min: 8, max: 20), the average length of the menstrual cycle was 29.41 ± 5.99 days (min: 15, max: 120), and the average duration of menstrual bleeding was 5.76 ± 1.53 days (min: 2, max: 15). On average, students replaced their menstrual hygiene products 3.90 ± 2.92 times daily (min: 1-max: 24), the average number of menstrual hygiene products known by the students was 3.16 ± 1.77 (min: 1, max: 7), and the monthly average budget allocated for hygienic products was $2.91 ± 1.91 (min: 0.21, max: 13.04) based on exchange rates on June 19, 2023.

Among the participants, 73.1% had regular menstrual cycles, 76.3% reported moderate bleeding, and 94.3% used disposable sanitary pads. The menstrual hygiene products known to the students included disposable sanitary pads, disposable tampons, menstrual cups, reusable pads, and menstrual panties. Of the participants, 68.0% received information about the menstrual product from family and friends, 92.2% did not use any genital products. According to the students, an ideal menstruation product should be comfortable (29.9%), leak-proof (27.6%), odor-absorbing (10.3%), and healthy/hygienic (8.0%). Regarding the hygiene conditions of school restrooms, 97.4% reported the presence of trash cans, 84.4% noted the availability of soap, and 12.9% stated that toilet paper was provided. While 69.2% of the students had never experienced a genital infection, 58.1% expressed interest in education on genital hygiene (Table 1).

**Table 1:** – Findings on the menstruation and genital hygiene characteristics of the participating students – İzmir, Turkey, 2022.

Characteristics	Number	%
**Menstrual cycle**		
Regular	857	73.1
Irregular	315	26.9
**Bleeding Volume**		
Mild	84	7.2
Moderate	895	76.3
Heavy	193	16.5
**Menstrual Product Used**		
Disposable sanitary pad	1105	94.3
Tampons	28	2.4
Menstrual cups	22	1.8
Reusable sanitary pad	3	0.3
Period panties	2	0.2
Others	12	1.0
Known menstrual hygiene products*		
Disposable sanitary pad	1163	99.2
Disposable tampons	694	59.2
Menstrual cups	600	51.2
Reusable sanitary pad	481	41.0
Period panties	448	38.2
Reusable tampons	237	20.2
Natural sea sponge	22	1.9
**Person/place from whom/which information about the menstrual product used was obtained**		
Family, friend	797	68.0
School	197	16.8
Social media	108	9.2
TV/internet ads	52	4.4
Health professional	18	1.6
**Genital Product use**		
Nothing	1081	92.2
Genital/Intimate hygiene solution	67	5.7
Whitening cream/gel	15	1.3
Genital area / Intimate deodorant spray	9	0.8
**Features of an Ideal Menstruation Product**		
Comfortable	351	29.9
Leak proof	323	27.6
Absorbing odor	121	10.3
Healthy/hygienic	94	8.0
Non-irritating/anti-allergic	82	7.0
Natural/Vegan	75	6.5
Economic	54	4.6
Reusability	7	0.6
Long-lasting	7	0.6
Unspecified	58	4.9
Hygiene in school toilets**		
There is a trashcan.	1141	97.4
Soap is available.	978	83.4
Toilet paper is available.	151	12.9
**Having Developed Genital Infection**		
Yes	361	30.8
No	811	69.2
**Willing to be Educated About Genital Hygiene**		
Yes	681	58.1
No	491	41.9
**Total**	**1172**	**100**

*Question with multiple answers.

**Students who answered as “yes”.

The mean scores obtained by the students from the GHBS and its subscales were as follows: overall GHBS: 90.38 ± 9.98 (min: 35, max: 115), General Hygiene Habits subscale: 45.76 ± 5.42 (min: 20, max: 60), Menstrual Hygiene subscale: 33.29 ± 4.11 (min: 8, max: 40), and Awareness of Abnormal Findings subscale: 11.33 ± 2.74 (min: 3, max: 15) (Table 2).

**Table 2:** Mean Scores obtained from the overall Genital Hygiene Behaviors Scale (GHBS) and its subscales – İzmir, Turkey, 2022.

	$\bar{X}$ ± SD	Min.	Max.	Cronbach’s α
General Hygiene Habits	45.76 ± 5.42	20	60	0.69
Menstrual Hygiene	33.29 ± 4.11	8	40	0.65
Awareness of Abnormal Findings	11.33 ± 2.74	3	15	0.76
GHBS Total	90.38 ± 9.98	35	115	0.81

A comparison of the students’ demographic characteristics and their GHBS scores revealed statistically significant differences in mean scores based on several factors, including year in school, marital status, perceived income status, attending a health-related department, willingness to receive training on menstruation and genital hygiene (p < 0.05). According to the results of the post-hoc Tukey test performed to determine from which sociodemographic characteristics the differences stemmed, first-year students had lower overall GHBS scores compared to second, third, fourth, and fifth-year students. They also scored lower in the Menstrual Hygiene and Awareness of Abnormal Findings subscales compared to second-year students, and lower in the General Hygiene Habits subscale compared to third-year students. Additionally, students who reported that their income was lower than their expenses had lower overall GHBS scores compared to those who reported equal or higher income levels. Students in health-related departments scored significantly higher on the GHBS than those in other departments (Table 3).

**Table 3:** Distribution of scores obtained from the genital hygiene behaviors scale according to various variables – İzmir, Turkey, 2022.

Variables			GHBS			General Hygiene Habits			Menstrual Hygiene			Awareness of Abnormal Findings		
Year in school	N	%	X ± SD	F	p	X ± SD	F	p	X ± SD	F	p	X ± SD	F	p
1	414	35.3	88.57 ± 10.04	5.564	**0.000**	45.01 ± 5.38	3.111	**0.015**	32.73 ± 4.07	3.934	**0.004**	10.81 ± 2.75	6.551	**0.000**
2	359	30.6	91.40 ± 9.25			46.31 ± 5.23			33.83 ± 3.92			11.52 ± 2.64		
3	210	17.9	91.27 ± 10.57			46.24 ± 5.64			33.40 ± 4.29			11.55 ± 2.84		
4	168	14.4	91.67 ± 10.40			46.04 ± 5.58			33.50 ± 4.31			11.92 ± 2.65		
5	21	1.8	89.80 ± 6.23			45.76 ± 4.57			32.28 ± 3.18			11.47 ± 2.74		
**Marital status**				**t**	**p**		**t**	**p**		**t**	**p**		**t**	**p**
Single	1149	98.0	90.27 ± 10.04	2.858	**0.004**	45.70 ± 5.40	–2.588	**0.010**	33.24 ± 4.11	–2.551	**0.015**	11.31 ± 2.74	–1.633	0.103
Married	23	2.0	96.26 ± 9.95			48.65 ± 5.49			35.34 ± 3.45			12.26 ± 2.68		
**Income status**				**F**	p**		**F**	**p**		**F**	**p**		**F**	**p**
Income less than expenses	500	42.7	89.29 ± 10.56	5.625	**0.004**	45.24 ± 5.64	4.803	**0.008**	35.06 ± 4.27	1.315	0.269	10.97 ± 2.94	7.69	**0.000**
Income equal to expenses	563	48.0	91.07 ± 9.38			46.02 ± 5.19			33.43 ± 4.00			11.61 ± 2.48		
Income more than expenses	109	9.3	91.88 ± 9.82			46.77 ± 5.38			33.55 ± 3.92			11.56 ± 2.90		
**Attending a health-related department**				**t**	**p**		**t**	**p**		**t**	**p**		**t**	**p**
Yes	721	61.5	91.34 ± 9.91	4.197	**0.000**	46.21 ± 5.46	3.629	**0.000**	33.51 ± 3.94	2.357	**0.019**	11.62 ± 2.57	4.536	**0.000**
No	451	38.5	88.85 ± 9.91			45.03 ± 5.29			32.93 ± 4.34			10.88 ± 2.94		
**Menstrual cycle**				**t**	**p**		**t**	**p**		**t**	**p**		**t**	**p**
Regular	857	73.1	90.77 ± 9.70	2.176	**0.030**	45.87 ± 5.38	1.212	0.226	33.40 ± 3.93	1.501	0.134	11.49 ± 2.66	3.277	**0.001**
Irregular	315	26.9	89.34 ± 10.66			45.44 ± 5.51			32.99 ± 4.56			10.90 ± 2.89		
**Having developed genital infection**				**t**	**p**		**t**	**p**		**t**	**p**		**t**	**p**
Yes	361	30.8	90.30 ± 10.13	0.191	0.849	45.46 ± 5.52	–1.234	0.218	33.24 ± 4.22	–0.231	0.817	11.58 ± 2.76	2.093	**0.037**
No	811	69.2	90.42 ± 9.92			45.89 ± 5.37			33.30 ± 4.06			11.22 ± 2.72		
**Willing to be educated about genital hygiene**				**t**	**p**		**t**	**p**		**t**	**p**		**t**	**p**
Yes	681	58.1	89.33 ± 9.79	4.273	**0.000**	45.24 ± 5.34	–3.823	**0.000**	32.92 ± 4.04	–3.604	**0.000**	11.16 ± 2.64	–2.550	**0.011**
No	491	41.9	91.84 ± 10.07			46.47 ± 5.46			33.79 ± 4.15			11.57 ± 2.85		

## DISCUSSION

Menstruation is a physiological condition experienced by every healthy woman, with menarche typically occurring between the ages of 10 and 16. In the early years of menstruation, young people’s knowledge of menstrual hygiene tends to be low, and their healthy hygiene behaviors may be inadequate, which is understandable^(14)^. In this study, the mean age of the participants was 21.01 ± 3.03 years (min: 17, max: 50), and their mean age of menarche was 12.90 ± 1.31 years (min: 8, max: 20), indicating that they have had nine years to develop improved hygiene behaviors. One of these behaviors is the frequency of changing menstrual products based on the volume of menstrual flow, which is important for preventing infections. In this study, participants reported changing their menstrual products an average of 3.90 ± 2.92 times per day. Several studies have reported that menstrual products are usually changed 2-4 times per day^(15,16)^. The higher frequency observed in this study may be attributed to the fact that the participants were university students, many of whom were studying health-related subjects, as opposed to secondary or high school students from countries with lower socioeconomic status as in other studies^(4,11,12)^.

The monthly average budget allocated by the students for menstrual products was $2.91. Given that nearly half of the participants relied on repayable government loans or scholarships, which are quite limited in amount, the cost of menstrual products is a significant factor when making purchasing decisions. A report by the United Nations Population Fund (UNFPA) titled “Menstrual Hygiene Management for Refugee Women and Girls in Turkey” highlights that five out of ten women and four out of ten girls are unable to afford menstrual products they need due to high costs. This situation, known as “Period Poverty,” leads to increased stress and fear, as well as reduced well-being^(17)^.

In terms of product preferences, 94.3% of the students indicated that they preferred disposable sanitary pads, primarily because they found them comfortable (29.9%) and leak-proof (27.6%). In Turkey, television remains a popular form of entertainment^(18)^, and sanitary pads are frequently advertised, while other menstrual products receive little to no media exposure. The media plays a crucial role in shaping public health perceptions, as it can quickly reach and influence large audiences. For instance, a study found that advertising strategies affect female consumers’ purchasing behaviors^(19)^. In countries with poor economic conditions, reusable menstrual products tend to be more common than disposable ones^(16,20)^. However, in cultures where virginity is highly valued, sanitary pads are more commonly used than products like menstrual cups or tampons^(21)^. Cultural norms, access to menstrual products, and the cost of these products influence women’s choices.

In this study, 68.0% of the students reported receiving information about menstrual products from family members or friends, highlighting the traditional structure of society. Since menstruation is often regarded as a taboo subject, girls tend to feel ashamed and turn to their closest relatives for guidance. This reliance on non-expert sources, such as mothers and friends, can perpetuate incomplete or outdated practices and lead to the continued use of products recommended by these individuals^(22)^. The literature review revealed that mothers are the primary source of information about menstrual products for their daughters, followed by friends, relatives, and teachers, with health professionals being the least consulted group^(7,15,16,20,23)^.

Menstrual products known by the participants in this study included disposable sanitary pads (99.2%), disposable tampons (59.2%), menstrual cups (51.2%), washable tampons (20.2%), and natural sea sponges (1.9%). In a similar study conducted in Turkey, 97.2% of women were aware of sanitary pads, 67.6% had heard of tampons, and 58.3% were familiar with menstrual cups. In that study, 76.0% of the participants used sanitary pads, 11.6% used tampons, and 29.4% used menstrual cups^(24)^. While environmentally friendly, organic products are more commonly known and used by women with higher education levels or those living in developed countries, women in underdeveloped regions often face challenges accessing healthcare and information. Consequently, they may resort to using cloth or even newspaper in place of disposable sanitary pads^(7,8,11,20)^. The current study’s results, where disposable sanitary pads were the most widely known and used product, may reflect the cultural significance of virginity in unmarried women, a concept that retains importance in Turkish society.

In the present study, 92.2% of the participants stated that they did not use any genital products for hygiene. The additional cost of such products likely contributes to this trend, as they represent an extra financial burden on top of menstrual product expenses. Studies on genital hygiene show that soap and water are the most commonly used methods for cleaning the genital area^(15,25)^, and this finding aligns with the literature. In the present study, approximately one-third of the students (30.8%) reported having experienced genital infections, and more than half of them (58.1%) expressed a willingness to receive education on hygiene. Obtaining information about menstruation from non-medical sources like family or friends, combined with insufficient disinfection resources in public spaces such as school toilets and dormitories, as well as improper hygiene practices, creates conditions that increase the risk of infection. Previous studies conducted among students in Turkey have revealed that the prevalence of urogenital infections ranges between 25% and 50%^(22,26)^. Considering that students often rely on family members for information, educational interventions targeting students may also positively impact their families by providing accurate knowledge.

The average GHBS score of the participants was 90.38 ± 9.98 (min:35-max:115). In studies involving students, the average overall GHBS ranged between 85.3 ± 10.1 and 95.50 ± 9.42^(22,27,28,29)^. The average GHBS scores in this study are consistent with those observed in studies conducted only in the health-related departments. Statistically significant relationships were identified between GHBS scores and factors such as year in school, marital status, perceived income status, attending a health-related department, willingness to receive training in menstruation and genital hygiene. As education level, age and income level increase, women’s level of menstrual hygiene-related knowledge, their ability to access hygienic products, and their purchasing power increase, and they develop healthy hygiene behaviors^(7,15,26)^.

The present study has several limitations. First, since it is a cross-sectional study, data were collected over a specific period, which may not have reflected changes in the variables investigated over time. The data collection link was sent to students in all the faculties via e-mail, but higher participation was observed among students attending health faculties, which could be due to the study’s relevance to there area of study. In addition, since students’ genital hygiene behaviors were self-reported, there might be a reporting bias.

## CONCLUSION

The results of this study demonstrated that the participants exhibited good genital hygiene behaviors. In addition, their awareness of general hygiene, menstrual hygiene, and abnormal findings, which are components of genital hygiene behaviors, was also at a good level. The majority of the participants used disposable sanitary pads as menstrual products and that they frequently replaced them. While they mostly consulted their families and friends to obtain information about menstrual products, they rarely consulted health personnel, and more than half of them wanted to receive training on genital hygiene. Although the participants demonstrated relatively good genital hygiene behaviors, approximately one third of them had genital infections, indicating a need for training on hygiene practices and health implications. Considering that menstrual products they used most were disposable sanitary pads, educating students about environmentally friendly recyclable menstrual products and encouraging them to use such products may be beneficial both ecologically and economically. In addition, it will be beneficial to organize genital and menstrual hygiene education programs at regular intervals led by university health teams help female students adopt behaviors that improve their reproductive health. It is also recommended that new health policies be developed to make menstrual products more accessible to young people.
